# Paraganglioma in the posterior mediastinum: a case report

**DOI:** 10.1186/s12872-020-01752-2

**Published:** 2020-11-23

**Authors:** Jong-Ho Nam, Jong-Seon Park, Joon Hyuk Choi

**Affiliations:** 1 Division of Cardiology, Department of Internal Medicine, Yeungnam University College of Medicine, 170, Hyeonchung-ro, Nam-gu, Daegu, 42415 Republic of Korea; 2 Department of Pathology, Yeungnam University College of Medicine, Daegu, Republic of Korea

**Keywords:** Paraganglioma, Pheochromocytoma, Mediastinum

## Abstract

**Background:**

Paragangliomas are tumors that arise from extra-adrenal chromaffin cells. Herein, we present a rare case of a functional paraganglioma in the posterior mediastinum.

**Case presentation:**

A 36-year-old man presented with paroxysms of chest pain and headache. At presentation, the patient’s blood pressure was 190/120 mmHg. Chest computed tomography and magnetic resonance imaging revealed a left paravertebral mass in the posterior mediastinum. ^123^I-metaiodobenzylguanidine scanning revealed focally high tracer uptake in the left paravertebral area. The metanephrine level in the urine was elevated, confirming a rare, catecholamine-producing, functional paraganglioma in the posterior mediastinum. Before surgery, the patient was prepared by orally administering α- and β-adrenergic blockers. The mass was then resected via a lateral thoracotomy. The metanephrine level in urine was normal 24 h after surgery.

**Conclusions:**

Paragangliomas in the posterior mediastinum are very rare, but more than half of all cases are functional. The associated symptoms are curable with complete resection, and long-term follow-up for recurrence is important.

## Background

Paragangliomas are tumors that arise from extra-adrenal chromaffin cells. Paragangliomas can produce catecholamines and may cause various symptoms including headache, palpitations, and sweating. Accordingly, it is important to diagnose, localize, and remove paragangliomas, because the associated symptoms and hypertension are curable with resection and these tumors have malignant potential. Paragangliomas in the posterior mediastinum are rare, and more than half of the cases are functional [1, 2]. Herein, we report an unusual case of a functional paraganglioma in the posterior mediastinum of a 36-year-old man who underwent successful resection of the mass.

## Case presentation

A 36-year-old man was brought to the emergency room owing to chest pain and headache. He reported having intermittent episodes of chest pain, headache, and shortness of breath for 8 months. He had no other significant medical or family history and was not receiving any medication. At the time of presentation, the patient’s blood pressure was 190/120 mmHg and heart rate was 120 beats per minute (bpm). Electrocardiography revealed sinus tachycardia with a heart rate of 111 bpm. Routine laboratory test results including the cardiac troponin level were within the normal limits.

**Fig. 1:**
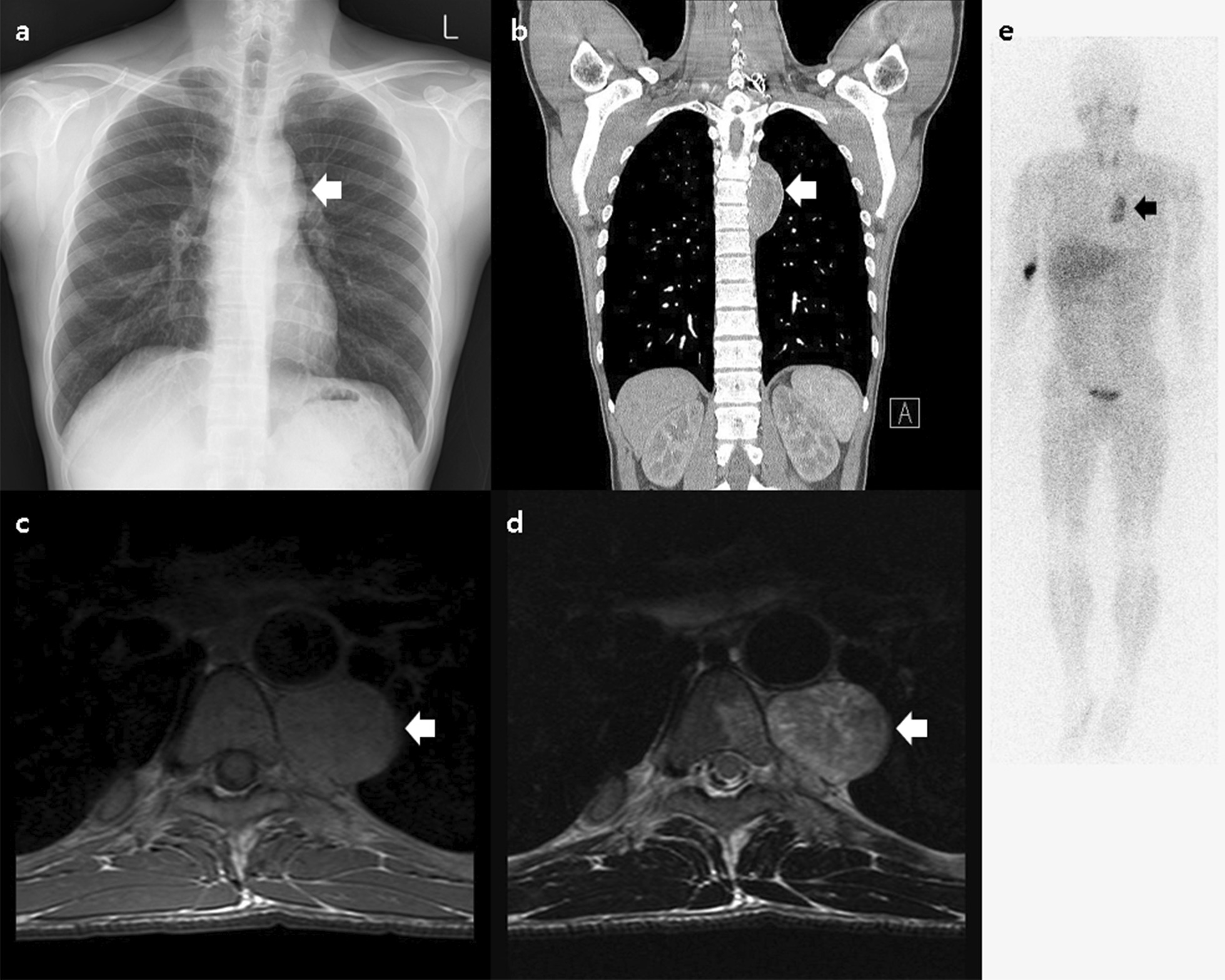
**a** Chest radiograph showing a mass-like lesion (white arrow) near the left hilum. **b** Coronal section of a contrast-enhanced chest computed tomography showing a 7-cm heterogeneous mass (white arrow) on the left side of the vertebra (T4-6 level). **c** and **d** Magnetic resonance imaging revealed that the paravertebral mass (white arrow) had intermediate signal intensity on the T1-weighted axial image (**c**) and high signal intensity on the T2-weighted image (**d**). **e** An ^123^I-metaiodobenzylguanidine scanning showing focally high tracer uptake in the left paravertebral area (black arrow)

Chest radiography revealed a mass-like lesion near the left hilum (Fig. 1a). On chest computed tomography (CT), a 7-cm heterogeneous mass was observed on the left side of the vertebra (Fig. 1b). T2-weighted magnetic resonance imaging (MRI) revealed that the paravertebral mass had a high signal intensity (Fig. 1c, d). ^123^I-metaiodobenzylguanidine (^123^I-MIBG) scanning revealed focally high tracer uptake in the left paravertebral area (Fig. 1e). On biochemical testing of urine, the patient had elevated levels of metanephrine (4.2 mg/day; normal range < 0.8 mg/day), norepinephrine (4946 μg/day; normal range 15–80 μg/day), and vanillylmandelic acid (26.6 mg/day; normal range 0–8 mg/day), confirming the diagnosis of a catecholamine-producing, functional paraganglioma in the posterior mediastinum.

**Fig. 2:**
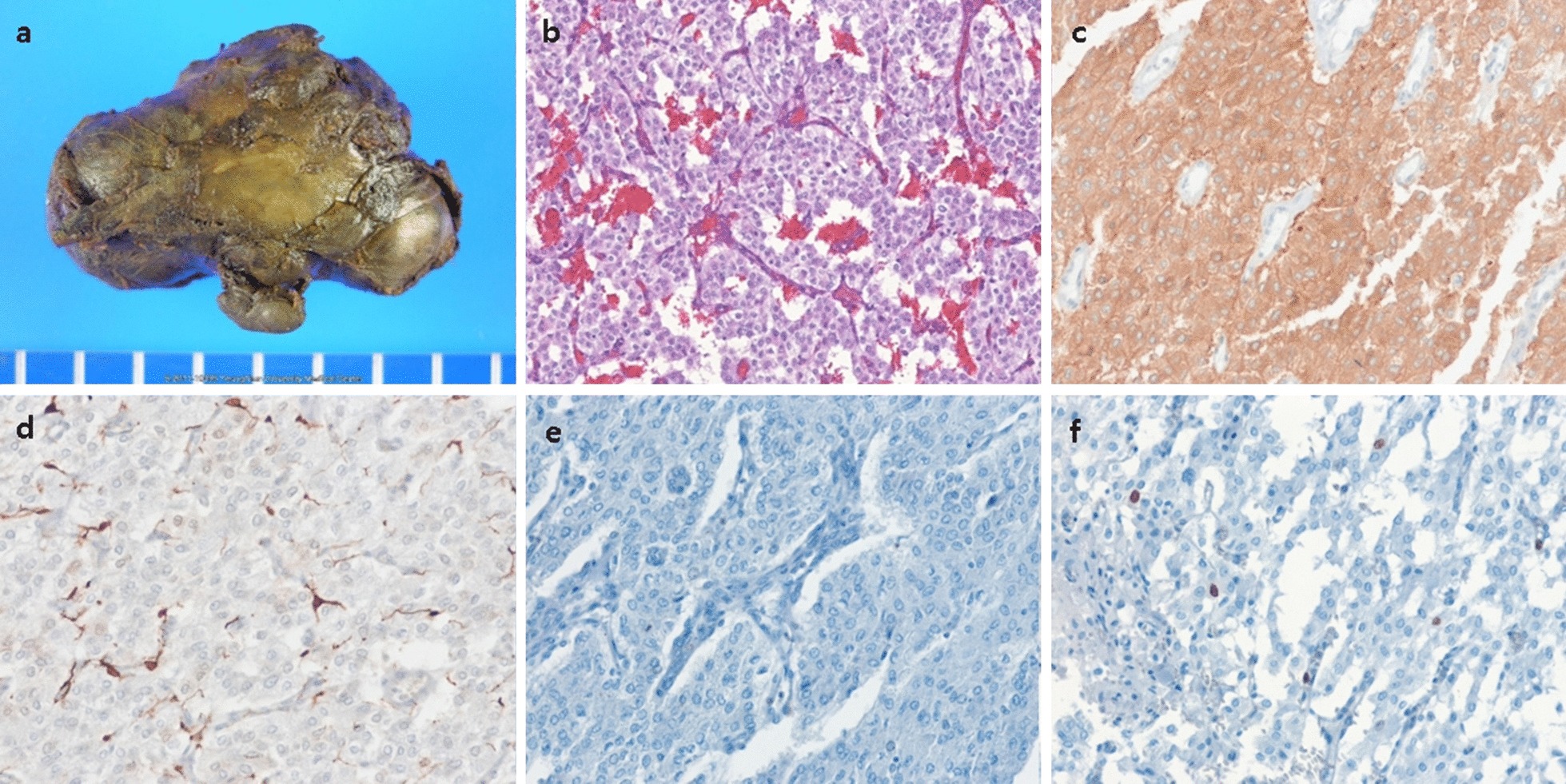
**a** The resected mass had a dark brown to gray surface and measured 6.5 × 4.0 × 3.0 cm (47.5 g). **b** On histologic examination of the hematoxylin and eosin stained specimen (magnification, 100 ×), polygonal tumor cells are observed to be arranged in a nested pattern (Zellballen structure). **c** Positive immunohistochemical staining for synaptophysin in the tumor cells (magnification, 200 ×). **d** Positive immunohistochemical staining for S-100 protein in the sustentacular cells (magnification, 200 ×). **e** Negative immunohistochemical staining for cytokeratins (magnification, 200 ×). **f** The positive rate of Ki-67 proliferation index was 1% (magnification, 200 ×)

The patient was prepared for surgery by orally administering the α-aderenergic blocker doxazosin (4 mg once daily) and the β-adrenergic blocker bisoprolol (2.5 mg once daily) for more than 1 month. The patient initially underwent thoracoscopic resection, which was later converted to lateral thoracotomy because the large-sized mass in the posterior mediastinum was tightly adherent to adjacent vessels and bled easily when manipulated and the patient had high blood pressure during the manipulation. The posterior mediastinal mass (6.5 × 4.0 × 3.0 cm) was successfully resected using lateral thoracotomy (Fig. 2a). The patient’s systolic blood pressure increased to more than 200 mmHg during resection, and temporarily dropped to 60 mmHg after resection, both of which were controlled with intravenous injections of a short-acting calcium channel blocker (nicardipine) and ephedrine. Histologic examination revealed the presence of Zellballen structures and absence of lymphovascular invasion and ganglion-like cells (Fig. 2b). On immunohistochemical staining, the mass tested positive for synaptophysin and S-100 protein (Fig. 2c and d) and negative for cytokeratins (Fig. 2e). The Ki-67 proliferation index was 1% (Fig. 2f).

After surgery, the patient’s blood pressure was stabilized at 100/60 mmHg and the heart rate was 85 bpm; hence, the administration of adrenergic blockers was stopped. Biochemical testing revealed that the metanephrine level in urine returned to normal (1 mg/day). The patient was asymptomatic and normotensive during follow-up.

## Discussion and conclusions

We demonstrate diagnosis of a rare, functional paraganglioma in the posterior mediastinum using biochemical testing, multimodality imaging, and histologic examination with successful resection. This tumor is very rare, but it is important for clinicians to suspect, diagnose, and resect it because it has malignant potential and can be fatal if it is undiagnosed and unresected.

Paragangliomas are neuroendocrine tumors that arise from chromaffin cells of the extra-adrenal tissue, while pheochromocytomas arise from chromaffin cells of the adrenal tissue [3]. Because paragangliomas and pheochromocytomas cannot be differentiated on the basis of the histologic findings, the anatomical location is used to distinguish between them [4]. The incidence of paragangliomas and pheochromocytomas is 0.6 cases per 100,000 person-years [4], and paragangliomas account for 10–15% of all chromaffin tissue-related tumors [5]. Only a few studies have evaluated the locations of paragangliomas. In a study by Erickson et al., most benign paragangliomas (69%, 204 of 297 tumors) were located in the head and neck area; paragangliomas in the mediastinum accounted for only 2% (6 of 297 tumors) of all benign paragangliomas [1].

Although the clinical presentation of paragangliomas can vary, most signs and symptoms—including hypertension, headache, palpitation, and sweating—are caused by excess catecholamine production and release, similar to the mechanism in pheochromocytomas. Measurements of plasma or urine metanephrines are the most sensitive tests for the diagnosis of catecholamine-producing, functional paragangliomas [3]. The paragangliomas in the head and neck are usually non-functional tumors. In contrast, only 17–43% of paragangliomas in the thorax, abdomen, and pelvis are non-functional [5]. In addition, more than half of the mediastinal paragangliomas are functional (66.7%, 2 of 6 tumors) [1].

If there is obvious biochemical evidence of paragangliomas, imaging studies should be performed to determine the location of the paragangliomas. CT is often performed for the initial localization of paragangliomas [3]. On CT, paragangliomas show enhancement after the administration of intravenous contrast media [6]. MRI usually reveals the paragangliomas to have intermediate signal intensity on T1-weighted images and high signal intensity on T2-weighted images, relative to the skeletal muscle. CT or MRI offer a high sensitivity, but limited specificity [3]. ^123^I-MIBG scanning offers a higher specificity and is useful for better assessment of the identification of detected masses by CT or MRI. ^123^I-MIBG scanning is also effective for the detection of additional multiple tumors.

Resection is the treatment of choice for paragangliomas. Before surgery, appropriate preoperative medical treatment is needed to control blood pressure and prevent hypertensive crisis during surgery. An α-adrenergic blocker is generally prescribed for at least 7 days, before surgery. A β-adrenergic blocker is prescribed for controlling tachycardia only after the α-adrenergic blocker is administered. Alternatively, intraoperative hypertension and bleeding can be potentially prevented by performing preoperative angiography and embolization of the vessels feeding the paragangliomas [7]. Rakovich et al. [8] have suggested that preoperative embolization should be considered to reduce the vascularity of the mediastinal paragangliomas, thereby facilitating surgical resection. However, Paul et al. [9] have suggested that tumor necrosis by preoperative embolization may induce an uncontrollable hypertensive crisis through catecholamine release. Considering these controversies and a lack of consensus in support of this approach for the mediastinal paragangliomas, we believe that an individualized approach is essential. Preoperative embolization may be reserved for highly vascularized and larger mediastinal paragangliomas that have been previously treated with an α-adrenergic blocker and cannot be excised because of hemodynamic instability [10, 11].

For resection of pheochromocytomas and paragangliomas, a minimally invasive approach such as laparoscopic resection is the standard for pheochromocytomas; however, endoscopic resection of paragangliomas is optional [12]. Because paragangliomas are more likely to be malignant and are frequently found in areas difficult for endoscopic resection, they are more likely to require open resection than pheochromocytomas [12]. The 2014 pheochromocytoma and paraganglioma guideline issued by the Endocrine Society also suggests open resection for paragangliomas [12]. The guideline further states that endoscopic resection can be perfomed for small, noninvasive paragangliomas in surgically favorable locations [12]. In our case, thoracoscopic resection was converted to lateral thoracotomy to limit bleeding and reduce hemodynamic instability as the large-sized paraganglioma was located along the thoracic aorta and firmly adhered to adjacent vessels.

The prognosis of paragangliomas is closely associated with the resectability and genetic profile [5]. Complete resection is the only curative treatment option. However, even after complete resection of paragangliomas, a significant proportion of patients experience recurrence. Erickson et al. reported that resection was not curative in approximately one-third of patients with benign paragangliomas (31%, 59 of 192) [1]. In a study by Brown et al., among 10 patients with mediastinal paragangliomas who underwent complete resection, late recurrence was noted in 2 patients [2]. Therefore, after surgery, all patients should undergo long-term follow-up with regular biochemical and imaging tests to identify recurrence.

More than 40% of patients with pheochromocytomas or paragangliomas carry germline mutations [4]. And it has been suggested that all patients with pheochromocytomas or paragangliomas should be considered for genetic testing, because metastasis and recurrence are highly probable in patients with germline mutations and the syndromes associated with pheochromocytomas and paragangliomas are also associated with other neoplasms, all of which require regular surveillance and treatment [3]. Clinical clues for genetic testing are a positive family history; young age; the presence of bilateral, multifocal extra-adrenal tumors; and the presence of other tumors. The patient reported herein was not tested for genetic mutations. Although he had no family history and no other lesions present on CT, MRI, and ^123^I-MIBG scanning, he was relatively young age and had extra-adrenal tumors, both of which are indications for genetic testing.

In conclusion, paragangliomas in the posterior mediastinum are very rare, but more than half of all cases are functional. The associated symptoms of these rare, functional tumors are curable with complete resection, and long-term follow-up for recurrence is important.

## Data Availability

Not applicable. All data supporting the conclusions are presented in the manuscript.

## Abbreviations

BPM: Beats per minute
CT: Computed tomography
MRI: Magnetic resonance imaging; ^123^I-MIBG scanning; ^123^I-Metaiodobenzylguanidine scanning
